# Social media as a data resource for #monkseal conservation

**DOI:** 10.1371/journal.pone.0222627

**Published:** 2019-10-23

**Authors:** Mark Sullivan, Stacie Robinson, Charles Littnan

**Affiliations:** 1 Joint Institute for Marine and Atmospheric Research, University of Hawaii at Manoa, Honolulu, Hawaii, United States of America; 2 Pacific Islands Fisheries Science Center, National Marine Fisheries Service, Honolulu, Hawaii, United States of America; Auburn University, UNITED STATES

## Abstract

The prevalence of social media platforms that share photos and videos could prove useful for wildlife research and conservation programs. When social media users post pictures and videos of animals, near real-time data like individual identification, sex, location, or other information are made accessible to scientists. These data can help inform researchers about animal occurrence, behavior, or threats to survival. The endangered Hawaiian monk seal (*Neomonachus schauinslandi)* population has only 1,400 seals remaining in the wild. A small but growing population of seals has recently reestablished itself in the human-populated main Hawaiian Islands. While this population growth raises concerns about human-seal interactions it also provides the opportunity to capitalize on human observations to enhance research and conservation activities. We measured the potential utility of non-traditional data sources, in this case Instagram, to supplement current population monitoring of monk seals in the main Hawaiian Islands. We tracked all Instagram posts with the identifier *#monkseal* for a one-year period and assessed the photos for biological and geographical information, behavioral concerns, human disturbance and public perceptions. Social media posts were less likely to provide images suitable for individual seal identification (16.5%) than traditional sighting reports (79.9%). However, social media enhanced the ability to detect human-seal interactions or animal disturbances: 22.1%, of the 2,392 Instagram posts examined showed people within 3 meters of a seal, and 17.8% indicated a disturbance to the animal, meanwhile only 0.64% of traditional reports noted a disturbance to the animal. This project demonstrated that data obtained through social media posts have value to monk seal research and management strategies beyond traditional data collection, and further development of social media platforms as data resources is warranted. Many conservation programs may benefit from similar work using social media to supplement the research and conservation activities they are undertaking.

## Introduction

The increase in availability of smartphones equipped with Global Positioning Systems (GPS) capabilities, high-resolution cameras, and internet connectivity [1] paired with the increase in social media usage, has inadvertently outfitted an immense network of people to potentially contribute to conservation science. Generally, social media is used to share information with a broad public audience and has been accepted as a productive way for scientists to engage the public in an outreach capacity [2]. The advent of tools such as hashtags, geo-tags, and other keyword identifiers means posts are increasingly easy to search and filter [3]. These identifiers enable conservation scientists to more easily mine data specific to a species or location and provide additional insight into human components of wildlife recovery and management.

Information gleaned from social media posts may be particularly beneficial for rare or cryptic species that are difficult to monitor across their range. Many species are too numerous and/or inhabit a range too large to be surveyed by scientists effectively resulting in many financial and logistical hurdles to overcome in order to monitor a population [4]. In addition to creating a stream of cost-effective data, social media is a great platform for citizen science because it provides an interactive learning experience where contributors can connect with other enthusiasts and provide valuable data to administrators [5].

The nature of social media creates unique opportunities to monitor public perceptions of and interactions with wildlife. Where humans and wildlife overlap, it is inevitable that interactions take place. These interactions can often be to the detriment of the animals and need to be monitored to inform management decisions for the benefit of the species [6, 7]. Human disturbance can have high cumulative impacts on wildlife whether it involves direct interaction or elevated environmental disruption such as noise [8]. In some cases interactions become elevated to human-wildlife conflict, increasing the risk to physical safety for both animals and humans [6]. Because negative perceptions of a species can increase the potential for human-wildlife conflict, it is important to periodically gauge public attitudes toward endangered species to guide public information and outreach strategies. Monitoring social media could be one way to gain perspective on public perceptions of certain species.

The endangered Hawaiian monk seal (*Neomonachus schauinslandi)* provides an excellent case study for the utility of social media in conservation science: rare and sparsely distributed, yet charismatic and attractive to social media users and the public in general. The current population of just 1,400 animals primarily inhabits the remote Northwestern Hawaiian Islands (NWHI) where populations have been systematically surveyed for over three decades [9]. A smaller population has recently re-established itself in the heavily human-populated main Hawaiian Islands totaling 231 seals in 2015 [9]. In the main Hawaiian Islands, National Oceanic and Atmospheric Administration (NOAA) scientists rely on public reports of seal sightings to most efficiently monitor this recovering population [10]. Many sighting reports come in through organized volunteer networks and a seal sighting hotlines. Yet, there are still many instances where social media may reveal seal sightings of conservation interests that would otherwise go unrecorded. These supplemental data could prove valuable to monk seal population monitoring as well as other facets of conservation research such as tracking animal disturbance and public perceptions of wildlife.

Monk seals occur across the main Hawaiian Islands utilizing beaches from the most popular to the most remote spanning >1,600 km of shoreline [11]. The sparse and widespread nature of this small population, makes them particularly challenging to monitor. Regular survey efforts are highly inefficient for agency biologists, but widespread public contributions greatly improve data collection. Island residents and visitors travel widely throughout the main Hawaiian Islands [12, 13], and these beach users often take pictures of the seals they encounter and post them on social media with hashtags such as #monkseal. By monitoring social media and filtering for hashtags, wildlife researchers can cover more ground virtually and access data from locations that are not surveyed by standard means [14]. Hawaiian monk seals may be relatively easy to identify individually from social media photos because many are tagged or possess unique scar patterns. Similar methods can apply to many wildlife species that can be identified in photographs [15, 16]. For example, like monk seals, Steller sea lions that have been instrumented, have entanglement scars, injuries, or other irregularities may stand out in photos and highlight management concerns [17].

As the monk seal population continues to grow in the heavily populated areas of the main Hawaiian Islands, seals are increasingly at risk of disturbance from human activity. Monk seals are particularly likely to encounter people as they haul out of the water for critical life history functions such as pupping, resting, thermoregulation, and avoiding predators. High levels of human disturbance can threaten seals and have been linked to historical monk seal population declines [18, 19]. The images shared through social media may reveal interactions between people and monk seals to provide an estimate of the prevalence and severity of human disturbance and inform strategies to reduce them.

The overarching goal of this project was to evaluate the utility of social media for informing species conservation, specifically exploring Instagram posts about Hawaiian monk seals. In order to determine the value of social media posts as a data source for population monitoring, we compared the frequency of obtaining individual animal identifications through social media versus traditional data collection methods. We hypothesized that seals and their locations may be harder to identify from photos posted on social media, as compared to sightings reports which are typically from trained volunteers and other relatively informed members of the public. Additionally, to assess the value of social media in capturing human disturbance of wildlife, we compared the frequency and degree of human disturbance depicted in posts to that collected through standard reporting channels. We hypothesized that relatively few beach-goers would be informed or observant enough to notice and report most disturbances to wildlife. Meanwhile social media users might inadvertently self-report disturbance to the subject of their photos. We expect that because public reporting systems require a high level of awareness and either self-reporting or community-policing, they are likely to be insufficient to accurately interpret the true level of disturbance inflicted on animals. Finally, we examine what other aspects of monk seal conservation and management can be informed through social media posts. If social media can benefit Hawaiian monk seal monitoring and recovery, it may prove to be a valuable tool for other wildlife research and conservation organizations worldwide.

## Methods

Ethics statement– All data for this study were obtained from the social media platform Instagram, and followed its’ terms and conditions for protecting their users. This project did not require Office of Management and Budget approval under the Paperwork Reduction Act because it did not involve any information collection, but was restricted to analysis of publicly available data.

### Social media data query

We focused this study on posts on Instagram, a mobile media sharing application, due to the ease of mining data and the visual format of the content [20]. Because Instagram requires the use of photographs or videos, all posts could be used to potentially identify monk seals or seal disturbance. Instagram uses hashtags, a word or phrase preceded by the hash or pound sign (#) used to identify specific topics. At the time of this study, Instagram had over 300 million active users [21] and numerous posts were labelled *#monkseal* every day. Our specific search criteria involved one year of posts from 1 October, 2014 to 30 September 2015. We searched for posts including the hashtag *#monkseal*. Only publicly available posts were searched because posts by private accounts could not be seen and would not comply with our ethics protocol. The search produced over 2,500 posts from which we excluded those that did not portray wild, live, Hawaiian monk seals. Examples of posts that we excluded were of captive seals, artwork, dogs, and people pretending to be seals. We also excluded posts depicting Mediterranean monk seals which were obvious because the seals and beaches look much different than those of Hawaii and the post was often written in a European language. These criteria yielded 2,392 posts depicting wild, live, Hawaiian monk seals.

### Population monitoring

Data collected from each post included: date, location, and Instagram profile name. When discernible, the individual seal’s size class (pup, juvenile, sub-adult, or adult) and sex were recorded. We also attempted to identify the seal by examining a variety of markings including flipper tags and bleach marks applied by scientists, natural markings, and unique scars. As a quality control measure, we required observation of at least three identifying marks to consider an animal “identified” (matching the criteria used in the formal monk seal sighting report database [22]). Most individuals in the relatively small main Hawaiian Islands population can be identified by these characteristics [23]. We recorded that the location of a post was identifiable by island or beach if it was geo-tagged by the poster or visually recognizable to our data collectors.

### Human-wildlife interaction

**Fig 1:**
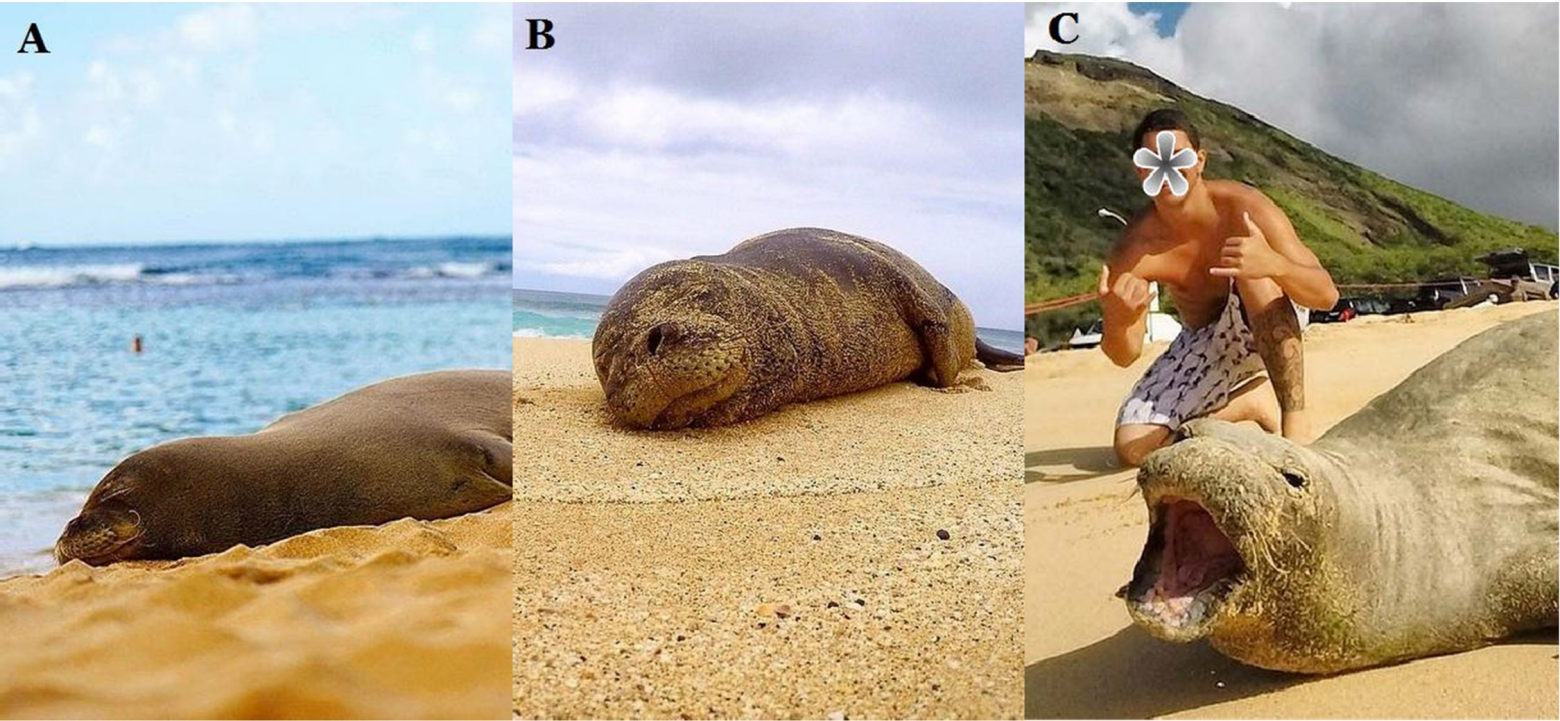
Photo characteristics can be used to judge distance at which photo was taken. A) The blurry foreground and background suggest a zoom lens was used from > 3-m away from the subject. B) All elements are in focus suggesting the use of a wide-angle lens taken from < 3-m from the subject. C) A person < 3-m from a seal and the seal displaying signs of disturbance.

For the purposes of this paper, human-wildlife interaction describes the direct interactions between humans and wildlife, not conflict between humans about wildlife. Monk seals have proven to be susceptible to disturbance and managers recommend that people maintain a distance of at least 50 m from seals to minimize disturbance. We estimated the distance of the photographer from the seal in each post using the image’s depth of field to determine the amount of zoom used. As depicted in Fig 1, when the seal was in focus while the foreground and background were not, the photographer was likely far from the seal. However, if the seal, foreground and background were all in focus the shot was likely created with a wide-angle lens from a close distance to the seal. Finally, if a person was visible in the frame, we estimated their distance from the seal as well.

Distances were estimated to be either less than 10 feet, 10–50 feet, or more than 50 feet from the seal. We also determined whether there were signs of disturbance to the seal which could include minor disturbance (the seal looking at the camera, open mouth display) or major disturbance (moving away from the camera or individual in the frame).

### Statistical comparison of data sources

We evaluated the utility of social media data against traditional data streams by comparing the Instagram dataset to NOAA’s monk seal sighting database. We queried NOAA’s monk seal sighting database for all sighting reports from the study period (1 October, 2014 to 30 September 2015). First, to assess population monitoring data, we evaluated whether the proportion of individual animals identified or beaches identified differed between the data collected from Instagram versus the traditional seal sighting reports. Next, to assess human-wildlife interaction data, we evaluated whether the frequency of disturbance detection (either minor, major, or total) differed between the data collected from Instagram versus the traditional seal sighting reports. We used a z test (computed using prop.test in R [24]) to test for meaningful differences between proportions (based on a critical p value of 0.05)

### Additional conservation insights from social media

Scientists and managers spend considerable time tracking seals that are injured or demonstrating behaviors that might be ultimately harmful to seal or human safety. NOAA maintains a list of these “seals of concern” to guide management actions or interventions. We recorded instances where an Instagram post depicted a seal interacting with humans (such as a seal that was in a boat harbor and being provisioned), or a seal that had a visible wound. We cross-referenced these findings with NOAA’s seals of concern list for the time period of the project, and compared the rate at which seals of concern were identified by social media versus NOAA’s traditional database.

Captions and comments on Instagram posts were used to give a general indication of public perceptions shared about monk seals. We examined both the poster’s original caption, termed “Poster’s remarks” and the cumulative comments from others, termed “Commenters’ remarks”. We described photo captions and comments as positive, negative, or neutral based on content. Given the brief nature of many social media comments, we limited our analysis to these basic descriptors and a more detailed content analysis was beyond the scope of this study. We deemed posts positive if they included indicative terms such as “amazing, happy and special”. We deemed posts negative if they included indicative terms such as “damn things” and “get your own fish”. Judging whether a person’s comment is positive or negative was a subjective endeavor; therefore, any verbiage that was not clearly positive or negative was recorded as neutral.

## Results

### Population monitoring

We positively identified the individual seal in 16.5% (n = 396) of the total 2,392 posts, representing 29.4% of the main Hawaiian Islands population at the time of the study (68 of 231 individual seals). We were able to identify the beach location in 42.2% (n = 1,011) of posts. In another 30.4% (n = 728), we could identify the island. In contrast, the traditional seal sighting reports individually identified a significantly greater proportion of the seals seen during this project period (z test p value 2.2E-16); 79.99% of 7655 Hawaiian monk seal reports included the seal’s identity (Table 1). Similarly, the traditional sighting reports included location data significantly more often than social media posts (z = 56.73, df = 2391, p = <0.00001), island or beach were specified in 97.78% of 7,655 reports (Table 1).

**Table 1:** Social media data vs traditional data for identifying Hawaiian monk seals, their locations and human disturbance. We show the number of seal reports obtained through traditional and Instagram-based reports, and results of z tests comparing the proportions of reports with seals/beaches identified and disturbances noted.

Instagram vs Traditional data 10/1/2014–9/30/2015								
	Traditional	Percent	Instagram	Percent	P value (Z test)	Z	df	P value
Seal reports—total	7655		2392					
Individual seals identified	205		68					
Seal reports—seal identified	6123	79.99%	396	16.55%	2.20E-16	56.73	2391	<0.00001
Seals of Concern	31	0.40%	14	0.58%	0.3284	-1.16	2391	0.123
Seal reports—beach identified	7485	97.78%	1011	42.26%	2.20E- 16	65.6	2391	<0.00001
Human disturbance—total	49	0.64%	426	17.80%	2.20E- 16	-34.53	2391	<0.00001
Human disturbance—minor*	19	0.24%	407	17.01%	2.20E- 16	-35.56	2391	<0.00001
Human disturbance—major**	24	0.31%	19	0.79%	0.003029	-3.15	2391	0.00164

*Minor human disturbance includes the seal looking, mouthing or moving at least a body length.

**Major human disturbance involves the seal flushing to the water.

### Human-wildlife interaction

We found many posts depicting photographers or other people closer to seals than the recommended 50 m. The cumulative total of posts depicting people too close to the seal or animal disturbance was 40.05% (n = 958) of the 2,392 posts. The photographer was within 3 m of a seal in 17.55% (n = 420) of posts. A person other than the photographer was within 3 m of a seal in 4.68% (n = 112) of posts. Major disturbance is categorized as the seal moving away from the photographer or the seal flushing into the water and was observed in 0.79% (n = 19) of posts. Minor disturbance to the seals was observed in 17.01% (n = 407) of posts with 16.42% (n = 393) looking at the camera or another person in the frame and 0.58% (n = 14) showing an open mouth display. There was a significant (z = -34.53, df = 2391, p = 2.2E-16) discrepancy in the level of detected/reported disturbance of seals between the social media and traditional datasets, with disturbance detected over 20 times more frequently in social media posts (Table 1). Only 49 incidences of disturbance were identified in 7,655 traditional reports compared to the 426 incidences seen on the 2,392 social media posts.

### Additional conservation insights from social media

Public remarks in Instagram posts showed very low instances of negativity. The poster’s remarks were positive in 75.1% (n = 1,796). Negative posts comprised only 0.2% (n = 5) of the 2,392 total posts. Overall, 20.6% of posts were deemed neutral (n = 494) and the remaining 4.1% had no caption (n = 97). Commenter’s remarks were deemed positive on 1,250 posts, 6 were negative, and 1,136 were neutral or had no comment.

## Discussion

Our evaluation of the utility of social media for informing conservation found that Instagram, as an example of social media, was a valuable tool in endangered species research and monitoring. The quality of the images was sufficient to allow scientists to positively identify individual animals and add to the monitoring database. While we were impressed by the data obtained via social media, individual seals and locations were more readily identified in traditional sightings reports, supporting our first hypothesis. However, social media outperformed traditional reporting in our second area of evaluation, detecting human-wildlife interactions. In support of our second hypothesis, we found that photos posted on Instagram were far more likely to capture disturbance or interactions as compared to traditional sightings reports.

Our analysis of Instagram photos revealed the high potential for human disturbance when wildlife enthusiasts approach animals for a picture to post on social media. The instances of disturbance noted in this study (17.8% of posts), give managers a better understanding of the extent of disturbance that was vastly underestimated (0.64%) by traditional means. While many may lack the awareness or inclination to report a wildlife interaction or disturbance, photo-based social media provides the major benefit of recording interactions regardless of the awareness level of the participants, thus potentially removing a source of bias. The extremely low rate of disturbance reported by traditional means should be considered by wildlife managers to evaluate their system of capturing such data and setting management guidelines [25]. In addition to causing an immediate stress response in animals [26], human disturbance brings with it the threat of human injury or seal habituation. Habituation can progress into aggressive behaviors necessitating strong management actions such as displacing or translocating certain individuals [10].

Another beneficial aspect of social media was the ability to monitor animal sightings in near real-time, giving researchers and managers more frequent and timely information. This benefit is important because sometimes scientists may look for a specific individual that may be in need of rapid intervention. Roughly 30% of all Hawaiian monk seals are alive today due to NOAA interventions to help recover the species [27]. Some of these interventions were successful because of timely reporting by the public. Whether sick, injured, entangled, or aggressive towards people, some seals are of particular concern. These injuries or behaviors also may increase the probability of images of these animals being shared via social media and thus supplement reporting and help inform management decisions. Furthermore, a seal that is developing undesirable habits such as interacting with people may be noticed on social media before the seal becomes dangerous or people realize they should be reporting the issue to authorities. These behaviors often develop in the water with snorkelers and are rarely reported to NOAA by traditional means, but may be more likely posted on social media. Leveraging social media to supplement NOAA’s traditional data streams can enhance the response to seals of concern and help to inform us of the ultimate outcome of these situations.

Of the 31 seals of concern NOAA was tracking during the project’s time frame, 14 were seen on social media (53.8%). The data provided by social media allowed for real time reports on seals of concern regarding their current location, updates on their body/wound condition, or snapshots of their human interactions. Some images provided locations not previously recorded for a particular seal. These additions to individuals’ ranges are valuable to researchers and managers providing information which can help inform future monitoring or intervention efforts. An example of this benefit is when seal R329 suffered a shark bite to her face and went missing for months until found on social media after swimming 70 miles to an island where there are usually only a handful of seal sightings reported annually. The post showed her wound healing well and gave NOAA a valuable update on her status as well as increasing the significance of the previously unsurveyed location where she was posted as seal habitat.

Understanding public perceptions is critical for addressing human-wildlife conflict [6]. It is possible that social media can supplement for traditional surveys or polls [28] and can be repeated over time to determine general trends and shifts in public perception. Our data was collected from people posting information on their public accounts which differs from the data of scientifically designed surveys. Our data showed a strong trend toward positive perceptions of monk seals and may reflect a hesitance to post negative or inappropriate content out of concern of negative feedback, public shaming, or even prosecution. This social pressure may limit the utility of using social media to accurately gauge public perception [29]. Furthermore, Instagram has been identified as a social media platform with an altered self-representation and high intent to deceive [30].

Social media offers the potential for two-way communication between wildlife managers and social media users. Comments could be posted by subject matter experts to gain further understanding and inspire a more positive sentiment towards certain species by providing general talking points and specific information on individual animals. Scientists could focus targeted outreach on the most negative or inaccurate statements, or posts illustrating inappropriate behavior around animals, or encourage greater reporting from individuals in areas that are rarely surveyed. The interest generated by social media posts about endangered wildlife presents an opportunity for managers to provide animal-specific information or suggest best practices to an audience that is likely to be receptive [29, 31].

Automation will be a key to maximizing data resources in publicly available social media posts. Collection and analysis can be assisted by social media management tools and analytical third-party dashboards [32]. Multi-platform studies could also be beneficial as each social media platform offers an additional group of users and different demographics that could provide different data or benefit from specific outreach messages.

We identified a number of limitations distinctly associated with social media which should be taken into consideration when using social media data. Social media data did not identify as many seals or seal locations as traditional data. This was expected because traditional data is comprised of people on the scene with a seal and most of the time, they know what beach they are at. They can also take more pictures of the seal and send them in to be analyzed. In this regard, social media data is considered to be supplemental to traditional data. Studies using data obtained from social media may not be perfectly replicable because data on social media platforms are not permanent. When an account is deleted, that account’s data is lost. Likewise, users can change their data by making edits to existing posts. Thus specifying the dates of data collection from social media platforms is crucial in reporting results. Multiple hashtag searches can be done for each species, be it the most common name, as we used, scientific name, or other. To thoroughly search social media for a species, researchers should consider multiple hashtag searches and cull duplicates accordingly. Bias is undeniable on social media and it is possible that the majority of users say positive things and refrain from being negative as they curate their online persona [33]. There may also be biases towards human disturbance because users may favor posting a picture that they feel is more interesting and evoking of likes and comments. This influence can lead to a user selecting a picture of an animal moving as opposed to sleeping or the user may pose closer to an animal than they would otherwise. Lastly, posters can be intentionally misleading about when or where a picture was taken. Users may post a picture from years ago and not say so. It is also common for users to tag a post with the wrong location. Though certain human components may affect data on social media, the volume of data presents a valuable resource, provided adequate quality controls are used.

## Conclusion

Communication via social media is valid and useful for endangered species conservation. Established organizations such as the National Parks Service with several million social media followers, have a tremendous potential to influence people worldwide [34]. We believe our results also demonstrate the utility of social media for conservation research. Social media will likely become an increasingly valuable and effective research tool as more users contribute to citizen science by posting high quality media and tagging it appropriately. Social media is also a good tool for mitigating wildlife disturbances with self-policing, whereby users share and learn what is socially appropriate and acceptable. Whether it is to collect or distribute information, the ubiquitous and cost-effective nature of the technology provides great opportunity for engagement in conservation science through social media.
